# DNA demethylation of neuronal cell death genes in depression

**DOI:** 10.1186/1756-8935-6-S1-P113

**Published:** 2013-04-08

**Authors:** Yurong Xin, Anne O’Donell, Yongchao Ge, Benjamin Chanrion, Andrew Dwork, Victoria Arango, J John Mann, Fatemeh Haghighi

**Affiliations:** 1 Department of Psychiatry, Columbia University and The New York State Psychiatric Institute, New York, New York, 10032, USA; 2 Department of Neurology, Mt. Sinai School of Medicine, New York, New York, 10032, USA

## Background

DNA methylation may play a role in the etiology of neuropsychiatric disorders through abnormal genomic methylation patterns regulating genes involved in brain development or physiology. In this study we explored the DNA methylation profile of depression in the prefrontal cortex because converging evidence from brain imaging and postmortem studies has implicated this region in depression neuropathology.

## Materials and methods

In order to better understand both the wild type genomic DNA methylation patterns and aberrant methylation events occurring in disease states we profiled DNA methylation patterns in human postmortem brains of 12 depressed and non-psychiatric controls using the methylation mapping and paired-end sequencing (Methyl-MAPS) method. Methyl-MAPS is an enzymatic-base method that can delineate the methylation status of greater than 80% of CpG sites genome-wide (Xin *et al*., doi: 10.4161/epi.6.11.17876). Overall, these data represent relatively unbiased coverage of the genome, including CpG-rich domains such as CpG islands and repetitive elements.

## Results and conclusions

Comparative analysis of DNA methylation patterns among depressed cases and controls revealed little variation at the transcription start sites (TSS) across all RefSeq annotated genes, but significant variations were detected proximal to the TSS (referred to as “CpG island shores”). We observed statistically significant methylation loss in CpG island shores in depressed cases compared to controls. These findings were replicated in purified neuronal cell populations. Using an independent sample of depressed cases and matched non-psychiatric controls, we isolated neuronal nuclei from the dorsal prefrontal cortex of 11 depressed cases and 11 controls. Due to limited quantities of neuronal DNA typically obtained from isolation of nuclei using fluorescence-activated cell sorting, we used the Illumina HumanMethylation450 BeadChip. DNA methylation differences in CpG island shores revealed that, of the CpG dinucleotides with significant methylation differences, >95% showed loss of methylation in depressed brains. The underlying mechanism involved in the loss of methylation in depression psychopathology is unclear. However, the global 5-hydroxymethylcytosine levels in neuronal DNA from the same sample specimens also showed a loss of hydroxymethylation in depressed brains compared to controls. Although these data were not statistically significant, they revealed an important trend in loss of hydroxymethylation and the possible mechanism for DNA demethylation in brains of depressed patients. Gene ontology analysis of genes with significant methylation differences (primarily loss of methylation) in depressed vs. controls identified a number of cellular functions. Of note, the fourth most significant gene set identified was involved in programmed cell death and 74% of the genes in this set were associated with neuronal cell death. These changes in methylation dynamics suggest a possible mechanism linking neuronal cell death associated with oxidative stress and inflammation in the depressed brain.

